# Pars basilaris size to estimate fetal and young infant age using forensic post mortem CT imaging

**DOI:** 10.1007/s12024-025-01039-y

**Published:** 2025-06-27

**Authors:** Wolf Schweitzer, Inga Siebke, Mattias Kettner, Stephan Bolliger, Carlo Tappero, Garyfalia Ampanozi

**Affiliations:** 1 Institute of Forensic Medicine, Universität Zürich, Zürich, Switzerland; 2 Zurich Forensic Science Institute, Zürich, Switzerland

**Keywords:** Anthropology, Forensic imaging, Pars basilaris, Fetal age estimation

## Abstract

Accurate determination of fetal or neonatal age is vital in forensic and medicolegal death investigations. The pars basilaris of the occipital bone, one of the earliest and densest ossification centers, is less susceptible to taphonomic alteration than other measurements, and exhibits predictable growth patterns. Utilizing post mortem computed tomography (PMCT), measurements of the pars basilaris – specifically its maximum length (ML) and maximum width (MW) – can be applied to validated regression models to estimate age. We retrospectively reviewed all fetal and stillbirth/neonate PMCT cases from our institution over the past eight years and identified nine cases with known or previously estimated ages. ML and MW of the pars basilaris were measured in thin maximum intensity projection reconstructions. Age predictions and 95% confidence intervals were calculated using published regression equations based separately on ML and MW. We also derived a combined model to yield a single age estimate with its corresponding confidence interval. In all nine cases, the predicted age intervals included the known or previously estimated age. The results indicate that pars basilaris biometry reliably estimates age using PMCT. Cases involving maternal conditions—such as diabetes, preeclampsia, and alcohol exposure—showed deviations from average pars basilaris growth but remained within statistical confidence limits. Pars basilaris biometry via standard PMCT protocols provides a straightforward method to approach fetal and early infant age estimation in forensic contexts. Although maternal and pathological factors can influence bone size, the combined ML/MW model is accurate within its 95% confidence bounds. Further research should validate these findings across diverse populations and investigate integration with additional growth markers.

## Background

Determining the age of a fetus or infant is crucial in forensic and medicolegal death investigations, including both individual cases and mass grave examinations [1]. When decomposition affects soft tissues, age estimation increasingly relies on osseous structures [2].

The pars basilaris of the occipital bone, a dense and early-forming structure in the occipital bone, is considered a relatively reliable anatomical feature for age estimation [3]. It is regarded as more resistant to taphonomic processes [4]. Across fetal age groups, the dimensions of the pars basilaris increase with age, and development of lateral angles and widening of the bone are observed [5]. A rule of thumb given in a previous study was that if the length of the pars basilaris exceeds its width, the age tends to be below $\sim 28$ weeks and if the width exceeds the maximum length, the age is likely to be over $\sim 5$ months postpartum [3].

Statistically validated regression models based on the pars basilaris of the occipital bone, specifically its maximum length (ML) and maximum width (MW), may provide reliable age estimates [3, 6]. Alternative anatomical markers for age estimation include dental development stages or long bone measurements [7]. When forensic cases are scanned with post mortem computed tomography (PMCT), measuring the maximum length and width of the pars basilaris is straightforward using standard imaging software [8].

**Table 1:** Case-related data and results

Description	RD [cm]	ST [mm]	Age [wks]	PB ML [mm]	PB MW [mm]	AE lo ML [wks]	AE hi ML [wks]	AE lo MW [wks]	AE hi MW [wks]	AE lo C [wks]	AE hi C [wks]
1 Dead at birth with 44 weeks age, maternal iron deficiency and placental pathology [21, 22]. A: 32.	14.3	0.6	44	17	16.4	34.3	44.6	33.6	50.8	34.0	46.7
2 Dead after birth with 37 +6 weeks age, pre-eclampsia [23]. A: 31.	14.8	0.6	43	16.5	16.9	32.8	42.6	34.3	52.4	33.5	45.0
3 Dead at 24 weeks age, mother survived suicide attempt by fall from great height, no fetal or placental pathology. A: 28.	75	0.6	24	11.2	8.7	21.5	27.8	20.0	25.0	21.2	25.5
4 Dead at estimated 26-28 weeks age, maternal chronic alcoholism, fetus with dysmorphic features [24, 25]. A: 37.	9.8	0.6	27$^\dagger$	12.5	10.7	23.0	29.9	23.9	31.4	23.7	30.4
5 Suicide of mother with 21 weeks age; maternal depression; no fetal/placental pathology [27]. A: 29.	35.0$^*$/15.9	1 /0.6	21	9.9	7.5	18.3	23.7	19.0	23.4	18.7	23.6
6 Dead at estimated 28 to 32 weeks age, dysmorphic features; maternal alcoholism [24, 25]. A: 32.	21.8	0.6	30$^\dagger$	13.1	10.8	24.3	31.5	24.1	31.7	24.2	31.6
7 Natural death of mother due to COVID-19 with 27 weeks age [28]. A: 31.	43.7$^*$/19.5	1.0/0.6	34	16.0	15.3	31.4	40.8	31.9	46.9	31.6	44.8
8 Dead at birth with vascular transposition with 39 weeks age, maternal diabetes mellitus [29–31]. Nicotine history of 1-2 cigarettes/day during pregnancy. A: 32.	14.4	1.0/0.6	39	16.8	15.6	33.7	43.8	32.4	47.9	33.1	44.8
9 Natural death of mother due to gestosis with 37 weeks age [26]. A: 32.	49.2$^*$/27.9	1.0/0.6	37	15.8	14	30.9	40.1	29.6	42.3	31.4	42.6

$^\dagger$: age estimated at autopsy based on body measurements. $^*$: PMCT of mother with fetus in utero. Abbreviations: **A**: age of mother [y]. **RD**: reconstruction diameter of PMCT data. **ST**: slice thickness of PMCT data ($512 \times 512$ matrix). **PB**: pars basilaris ossis occipitalis. **ML**: maximal length of pars basilaris, **MW**: maximal width of pars basilaris. **AE**: Age estimates in weeks [wks], **C**: combined model, **lo**: lower boundary, **hi**: upper boundary of 95% confidence interval

This study applies a recently published method based on ML and MW of the pars basilaris [6] to all nine cases that were identified in our case database to investigate its practical applicability and the plausibility of the results.

## Methods and material

### Case series

We reviewed all fetal and neonatal deaths in our database for which post mortem computed tomography (PMCT) had been performed. Using keyword searches for "stillbirth", "neonate", and "fetus", we identified all relevant cases over an eight-year span. Our database contained only these nine cases. No separate control or study groups were defined. Table 1 summarizes each case’s estimated (based on other measurements) or documented age (per police investigation) alongside the PMCT reconstruction parameters. Gestational ages are distributed almost evenly between 21 and 44 weeks, based on the available records.

### PMCT and image reading

**Fig. 1:**
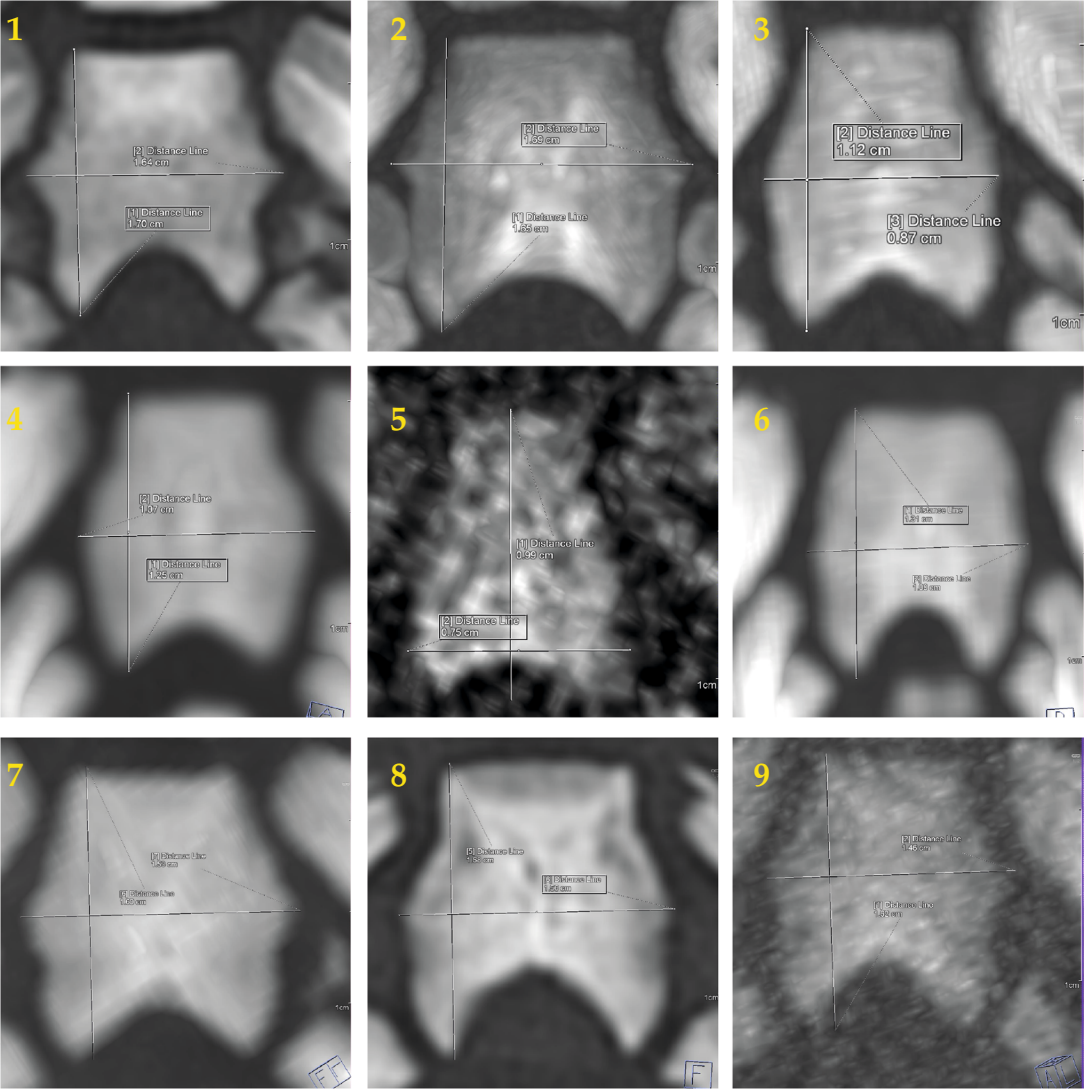
**Pars basilaris reconstructions in nine forensic cases.** In all nine cases, the pars basilaris of the occipital bone was reconstructed from PMCT scans using thin maximum-intensity projections (see Table 1 for details). In one case (case **3**), only a wide reconstruction field (diameter 75 cm) was available

**Fig. 2:**
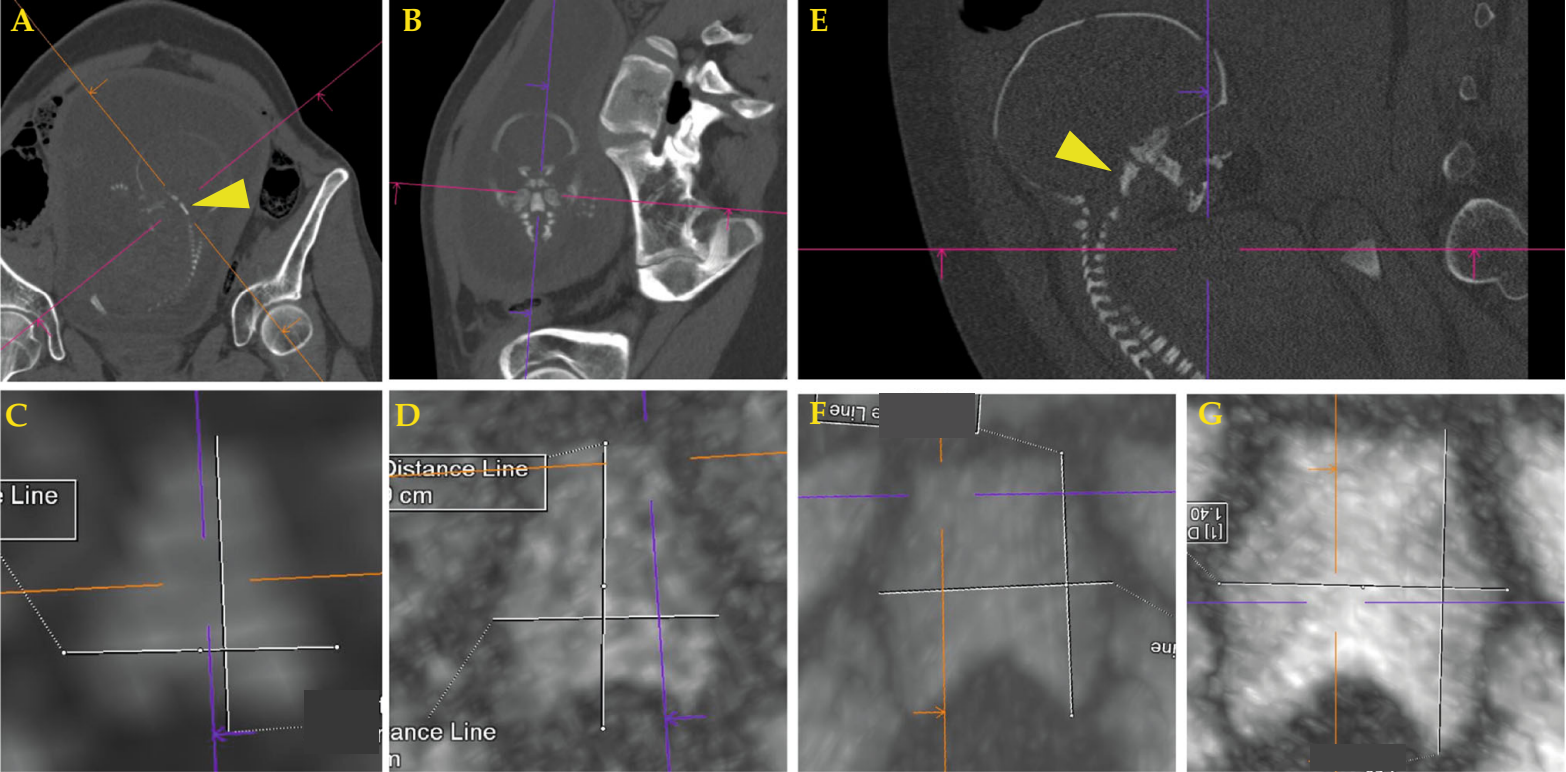
**Pars basilaris of occipital bone visualization from PMCT in utero.** The technique can be applied to fetuses also when PMCT was captured with the fetus in utero. From the cases that were evaluated (Table 1), case 5 (**A** and **B**: whole body PMCT with thorax/abdomen reconstruction of the mother, arrow indicates pars basilaris, **C**: pars basilaris reconstructed from that data, **D**: pars basilaris reconstructed from a focused PMCT data reconstruction of the fetus) and case 9 (**E**: whole body PMCT with thorax/abdomen reconstruction of the mother, arrow indicates pars basilaris **F**: pars basilaris reconstructed from that data, **G**: pars basilaris reconstructed from a focused PMCT data reconstruction of the fetus) show that even without dedicated protocol or higher quality data reconstruction, a useful visualization of the pars basilaris of the occipital bone may be obtained

**Fig. 3:**
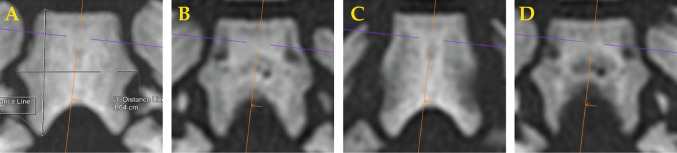
**PMCT reconstruction technique of pars basilaris of the occipital bone.** Thin maximum intensity projection (MIP) helps to properly visualize the maximal size extent of that bone as shown in **A**. Using simple multiplanar reconstructions (MPR), the maximal extent of that same bone is not just as reliable to reconstruct visually (**B**-**D**: various MPR levels through same pars basilaris as in A)

All cases were scanned following a standardized protocol using a Siemens Definition Flash CT scanner [9]. The data were evaluated using Siemens syngo.via software by a single reader, who measured the maximum length and maximum width of each pars basilaris of the occipital bone, as illustrated in Figs. 1, 2 and 3. Measurements were obtained without a detailed technical identification of bone margin thresholds [10]. Bone size measurement in CT may not require an additional 3D surface reconstruction step, when a thin maximum intensity projection (thin MIP) can be used instead [11]. Accordingly, all partes basilares were manually reconstructed using thin maximum intensity projection [12]. Relevant data pertaining to PMCT reconstruction and measurement results are presented in Table 1. With regard to this study cohort, measurement precision cannot be argued to suffer from a relatively small cohort size [13].

### Age estimation from PMCT measurements based on two published models

**Fig. 4:**
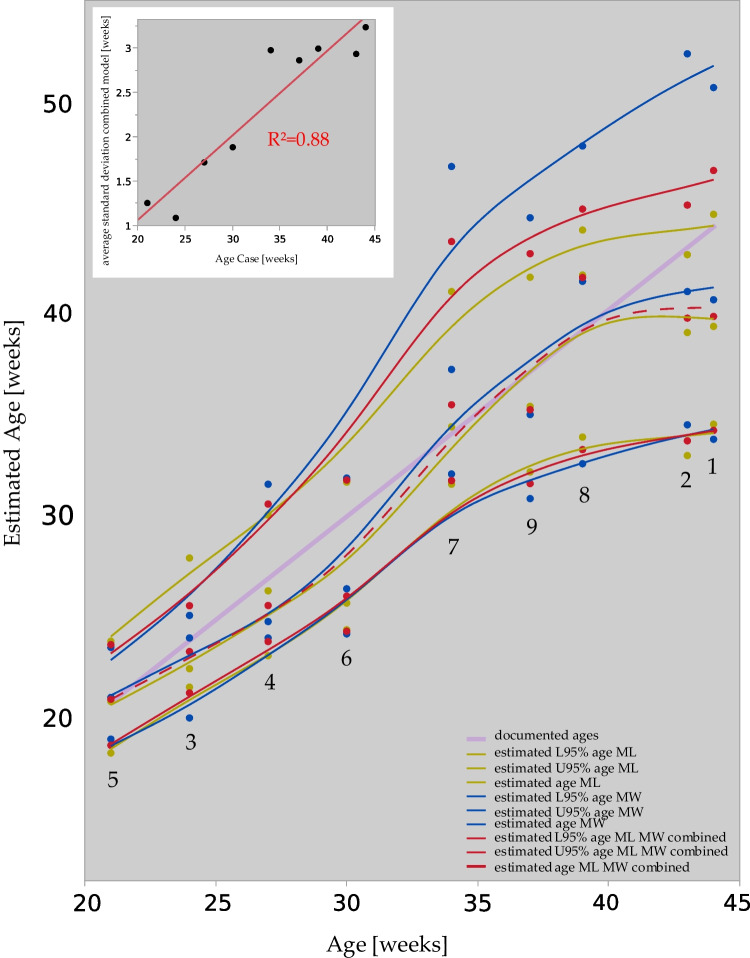
**Age estimates.** Combining both maximal width (**MW**, blue) and maximal length (**ML**, yellow) based statistical models results in a combined (red) model (method section for details). Mean estimates and lower (**L95%**) and upper (**U95%**) 95% confidence intervals as they relate to our own nine cases (as detailed in Table 1, data points annotated with case number reference). Documented ages in graph as purple line. – Inset diagram: age of the fetus correlated linearly with the standard deviations of the combined statistical estimates ($R^2 = 0.95$). – Curve interpolation as smoothed splines ($\lambda = 0.05$)

Age estimates and the corresponding 95% confidence intervals were obtained using the model equations as published by Niel and Adalian (2023) [6]. Two estimation methods were described in their study: one based on the maximal width and the other one on the maximal length of the pars basilaris ossis occipitalis. The resulting two single model estimates are shown in Fig. 4 as blue and yellow lines. According to that study, age can be estimated for individuals whose ML is between 7.25 and 21.07 mm and whose MW is between 4.41 and 21.35 mm [6]. Also, it is relevant to note that the model bases on 276 medical antemortem and post mortem computed tomography scans of fetuses and infants with normal development. Criteria included single pregnancy, absence of malformations or anomalies on imaging, a normal karyotype, and no maternal congenital diseases, diabetes, or hypertension. In addition, a combined model estimate was determined (see next section).

**Fig. 5:**
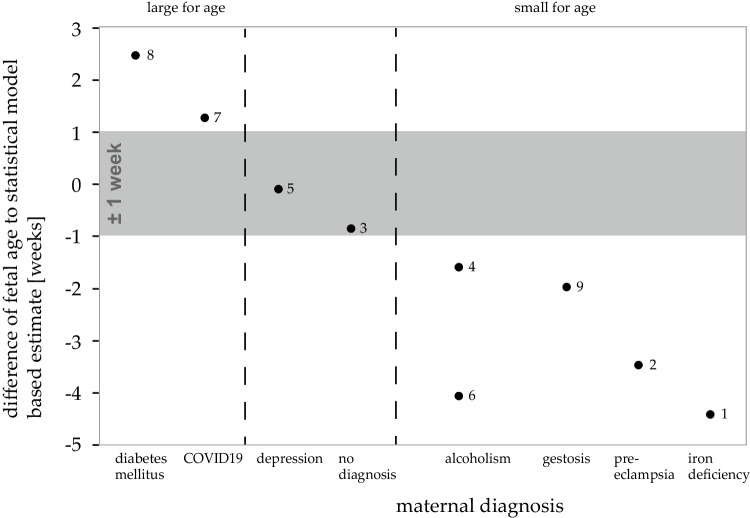
**Age estimates.** Correlation of maternal diagnoses (x-axis) and difference between estimated and actual age (y-axis). Case numbers as reference to Table 1

### Age estimation from PMCT measurements based on combined model of the two published models

Confidence intervals *c* as well as mean $\mu$ and standard deviation $\sigma$ are both related to the *Z*-score, see Eq. 1 [14].

1 $$\begin{aligned} Z = \frac{c-\mu }{\sigma } \end{aligned}$$

We can thus use Eq. 1 in order to arrive at a combined age estimate $E_c$ and to obtain 95% confidence lower ($L_c$) and upper ($U_c$) interval estimates. One might approach this by simply averaging the estimates [15]. As here, both MW and ML are correlated, a normal distribution for both probability densities was assumed. A combination of both the maximal width and maximal length derived age estimation ($E_{ML}$, $E_{MW}$) will allow to obtain a combined 95% confidence interval (there, $Z = \pm 1.96$) with its lower ($L_c$) and upper ($U_c$) bound once these conform to Eq. 2.

2 $$\begin{aligned} -1.96&= \frac{L_c-\mu _{E_{MW}}}{\sigma _{E_{MW}}} = \frac{L_c-\mu _{E_{ML}}}{\sigma _{E_{ML}}};+1.96\nonumber \\  &= \frac{U_c-\mu _{E_{MW}}}{\sigma _{E_{MW}}} = \frac{U_c-\mu _{E_{ML}}}{\sigma _{E_{ML}}} \end{aligned}$$

3 $$\begin{aligned} -2\cdot 1.96&= \frac{L_c-\mu _{E_{MW}}}{\sigma _{E_{MW}}} + \frac{L_c-\mu _{E_{ML}}}{\sigma _{E_{ML}}};+ 2 \cdot 1.96\nonumber \\  &= \frac{U_c-\mu _{E_{MW}}}{\sigma _{E_{MW}}} + \frac{U_c-\mu _{E_{ML}}}{\sigma _{E_{ML}}} \end{aligned}$$

Solving these equations, via step in Eq. 3, for $L_c$ and $U_c$ yields the expressions in Eq. 4:

4 $$\begin{aligned} L_c&\!=\! \frac{\mu _{E_{MW}} \cdot \sigma _{E_{ML}} + \mu _{E_{ML}} \cdot \sigma _{E_{MW}} - \sigma _{E_{MW}} \cdot \sigma _{E_{ML}} \cdot 2 \cdot Z }{\sigma _{E_{MW}} + \sigma _{E_{ML}} }; U_c\nonumber \\  &= \frac{\mu _{E_{MW}} \cdot \sigma _{E_{ML}} + \mu _{E_{ML}} \cdot \sigma _{E_{MW}} + \sigma _{E_{MW}} \cdot \sigma _{E_{ML}} \cdot 2 \cdot Z }{\sigma _{E_{MW}} + \sigma _{E_{ML}} } \end{aligned}$$

Given that the individual models showed only slight skewness (exponential regression for *ML* and reciprocal quadratic regression for *MW*) [6], the combined model confidence intervals were determined using separate upper and lower standard deviation estimates ($\sigma = \frac{| {CI}-\mu |}{1.96}$) [16]. The graphic results are plotted in Fig. 4. The logic behind identifying the confidence intervals for a combined model assuming normal distribution of the estimates is that for the mean value and standard deviation of each single model, the 95% confidence interval is identified as $\mu \pm (\sigma \cdot 1.96)$. So for two distributions, characterised by $\mu _1$, $\sigma _1$ and $\mu _2$, $\sigma _2$, respectively, these values in combination allow to solve for the newly estimated 95% confidence intervals. Combined age estimation $E_c$ based on both estimated age based on maximal width ($E_{MW}$) and maximal length ($E_{ML}$) as published [6] is thus approximated as follows (Eq. 5):

5 $$\begin{aligned} E_c = \frac{\mu _{E_{MW}} \cdot \sigma _{E_{ML}} + \mu _{E_{ML}} \cdot \sigma _{E_{MW}}}{\sigma _{E_{MW}} + \sigma _{E_{ML}}} \end{aligned}$$

The results of the combined model are illustrated in Fig. 4, with dots representing estimated age data points on the x-axis and effective age data points on the y-axis, and interpolated spline curves ($\lambda = 0.05$) for visualization.

### Statistics

Statistical evaluations were performed using Excel (Microsoft, Redmond, WA, USA) and JMP (SAS, Cary, NC, USA).

## Results

### PMCT resolution and age estimation

In three cases (cases 5, 7, 9), pars basilaris–based fetal age estimation was possible already based on the whole body PMCT of the mother. However, PMCT image quality was better when the fetus was scanned separately (Fig. 2). The technical details of PMCT scanning related to resolution (see Table 1) did not significantly correlate with the difference between the estimated and the documented age across these nine cases.

### Comparison of case documented age and statistical estimates

The size measurements of the pars basilaris for all cases (see Fig. 1) resulted in estimates that consistently included the previously known or estimated ages within the 95% confidence interval of the combined model, whereas no cases produced estimates outside the combined model’s 95% confidence boundaries. Detailed results are presented in Figures 4 and 5, as well as Table 1.

The ages of the fetuses correlated linearly with the standard deviation of the combined statistical estimates ($R^2 = 0.88$) (Fig. 4, inset diagram).

Within the 95% confidence interval, the combined model estimates resulted in partly overestimated and partly underestimated age results, when compared with the age given in our case documentations (see Fig. 5). Plausible reasons for that were, in our opinion, identified in the case specific information (see Fig. 5 and text below).

To explore the sources of the remaining underestimation and overestimation, we considered documented maternal conditions and further findings where available.

### Overestimated and underestimated results

An underestimated age suggests a pars basilaris smaller than average for normal development. Conversely, an estimate that is higher than the actual age points to a size of the pars basilaris that is above average for age. In two cases, age estimates were notably higher, and in five cases, they were considerably lower than predicted by the statistical models (see Fig. 5).

#### Correlates for potential differences between reported age and statistical age estimate

There are medical conditions that correlate with over- or underestimation of fetal age[17, 18]. These conditions may affect fetal growth, potentially leading to deviation from standard development [19, 20]. Some maternal diagnoses documented in our case files were associated with discrepancies between the age reported in case documentation and the age estimated by the combined statistical model (Fig. 5).

*Underestimation of age*

Case 1 – The fetus, that died during childbirth, was born to a mother with a history of treated iron deficiency. Laboratory tests did not identify low RBC or ferritin levels. Pedopathological examination revealed persistent extramedullary myelopoiesis and erythropoiesis in the liver. The placenta exhibited velamentous cord insertion, as well as chorioamnionitis and vasculitis of the chorionic plate vessels. These findings are consistent with intrauterine growth retardation [21, 22].

Case 2 – The childbirth was performed prematurely via cesarean section at 37 weeks due to maternal preeclampsia, characterized by hypertension and proteinuria. The newborn died six weeks later [23].

Case 4 – Stillbirth at estimated gestational age of 26-28 weeks, there was maternal chronic alcoholism, the fetus exhibited dysmorphic features [24, 25].

Case 6 – The fetus exhibited dysmorphic features, the mother was reported to intermittently consume 1–2 bottles of vodka per day, also during pregnancy [24, 25].

Case 9 – Natural death of mother due to gestosis with fetus at 37 weeks age [26].

*Correct estimation of age*

Case 3 – The fetus was dead at 24 weeks age while the mother survived a suicide attempt by fall from great height, where medical evaluation identified a complex pelvic fracture, retroperitoneal hematoma and a nonviable pregnancy. The mother had no reported underlying illnesses, although significant cultural and language barriers were noted. An emergency caesarean section was performed. No fetal or placental pathology was identified. The statistical age estimate was consistent with the predicted gestational age.

Case 5 – Maternal depression, suicide of mother with fetus at 21 weeks age; no fetal/placental pathology [27].

*Overestimation of age*

Case 7 – The mother died of a COVID-19 infection with extensive pulmonary artery thrombembolism while pregnant [28]. A request for a pedopathological autopsy of the fetus was rejected by the state attorney.

Case 8 – The fetus had a transposition of the large arteries of the heart, the mother suffered from diabetes mellitus [29–31].

### Technical aspects of CT reconstruction

As a technical illustration of the thin maximum intensity projection (MIP) that we would recommend to be used, a side-by-side comparison of thin MIP and normal multiplanar reconstructions (MPR) is shown in Figure 3. There, regular MPR images do not allow to measure maximal extent of the bone with the same reliable convenience as the thin MIP images do.

## Discussion

This study applies a model based age estimation method to nine medicolegal fetal death cases. Age estimates were obtained from bone measurements of the pars basilaris of the occipital bone that were fed into statistical models [6].

As that study by Niel and Adalian (2023) based its data on both antemortem and post mortem data, it can be assumed that their age estimation method developed on that basis may be used for living as well as dead fetuses or infants. The statistical context of our case series is that of a Swiss population with a statistically taller body stature than the French population on which the models were developed^1^. Regardless, the predicted 95% confidence intervals of our combined model consistently included the previously provided ages from case files in all nine cases. Notably, in our case series, the maximal lengths of the pars basilaris exceeded the maximal widths in every instance, consistent with prior research: this observation aligns with findings that the length of the pars basilaris typically surpasses its width until some time between the third and sixth postpartum months [3, 5, 6, 34].

We tested the referenced statistical method [6] and our combined model with just nine cases. With that, the question was raised how the result of our practical test into practical applicability and plausibility amounts to a statistical validation. We can assume a fit *F* of each of our nine cases into the combined model’s 95% confidence interval as being the result if pure chance, in a 50/50 scenario. We assume that each case fits the model randomly, and define that as hypothesis $$H_0$$. One can argue also that all of our nine cases are statistically unrelated. With that, the probability $$p (F | H_0) $$ of all nine cases being contained within the 95% confidence intervals by chance alone is $$p (F | H_0) = 0.5^9 = 0.002$$. Alternatively, we may assume as hypothesis $$H_1$$ that our combined model does not randomly but purposefully provide a fit *F* for each case within the confines of its 95% confidence interval. If we therefore assume each case to fit the combined model because it is tested, reliable, applicable and valid as we clearly expect it to do that each and every time, $$p (F | H_1) = 1^9 = 1$$. The likelihood *l* that it is by pure chance alone that all nine case age estimates are contained within the 95% confidence intervals of the combined statistical model, notwithstanding other considerations, thereby calculates as $$l = \frac{p (F | H_0)}{p (F | H_1)} = 0.002$$ or $$0,2\%$$.

The regression equations used in this study (see details in [6]) are (1) an exponential model for *ML*, in which estimated age increases exponentially, and, (2), a reciprocal quadratic model for *MW*, which peaks outside the stated range of applicability and then would yield further age estimates that decrease with increasing age. Only selected segments of each curve were applied in this model. Notably, neither curve fully models or represents the decelerating increase that characterizes pars basilaris growth. Fitting such equations to partial data suggests they may inadequately capture the underlying biological processes and risk overfitting the sample [35]. In contrast, pars basilaris growth is best described by a monotonic, decelerating function [3, 36].

Age estimation becomes less accurate with increasing age. A previous approach at defining a regression formula to estimate fetal age from pars basilaris dimensions described an increasing error with increasing age [37]. In this study, the ages of the fetuses correlated linearly with the standard deviation of the combined statistical estimates ($R^2 = 0.88$) (Fig. 4, inset diagram).

Collecting large representative fetal and infant data collections has been recognized as notoriously difficult [37]. Since this study is based on only nine cases, the sample size is clearly too small to draw robust statistical conclusions in a way that a new reliable statistical model emerges. These cases are not to be regarded as representative of a large or healthy population. They are limited forensic fetal or neonatal death examinations. While the underlying statistical model was designed to describe normal fetal development based on a French population [6], the unique circumstances including maternal and fetal conditions possibly prevalent in forensic or medicolegal cases may deviate from typical patterns [2, 38–41]. Specific forensic case related difficulties pertain to the isolated find of human remains of an infant or fetus. A high degree of fractures, unknown maternal illnesses and conditions, shrinkage in dry skeletal remains, or bone shrinkage in a burnt body all may further impede an accurate age estimation [42–44].

Differences between known and estimated age in individual cases may be plausibly explained in some of our nine cases. In order to be able to match known and estimated age against each other, it is relevant to note that the actual age has to be known. This data may not be available in forensic cases. In our study, two cases with small-for-age development could be attributed to fetal alcohol syndrome [24]. For tobacco and alcohol consumption, there even seems to be a dose-effect correlation [45]. Other maternal conditions correlating with small-for-age pars basilares included iron deficiency and placental pathology [21, 22], pre-eclampsia [23], and gestosis [26]. Conversely, two cases exhibited large-for-age pars basilaris measurements. One case was that of a maternal COVID-19 infection which may be associated with both intrauterine growth retardation and accelerated longitudinal growth [28, 46]. In the other case, maternal diabetes mellitus and transposition of the great arteries correlated with an apparent fetal growth acceleration [29–31].

Modelling normal growth on the basis of a general, healthy reference population is widely acknowledged as essential [47]. However, using only fetal or infant biometric measurements to estimate age may result in wrong estimates, particularly in heterogeneous subgroups, since growth trajectories are also shaped by genetic, environmental and health factors. Moreover, forensic cases may pose additional challenges as outlined above. This may appear to limit the model’s applicability across diverse forensic settings when in fact, correct age estimation may depend on more than biometric data of the fetus or infant. Conversely, a model defined by a relatively narrow statistical norm for healthy fetal development can be useful: our case series demonstrates that estimating gestational age by comparing individual measurements against this norm – and then integrating ancillary information (for example, confirmed chronological age or maternal health conditions, which may not be available at the time of examination) – yields relevant additional information.

Visually informed bone measurements may risk to yield lower values than threshold–based automated determinations. Precise CT density thresholds to match bone size may be difficult to generalize, however, as CT densities for all materials differ significantly across CT scanners [48]. A more reliable bone edge determination may therefore also have to use other factors than CT density, such as gradients [49]. Upon digitization, any single real feature requires a minimal resolution of 16 to 24 data elements in each dimension (pixel, voxel, 3D coordinate points) for an adequate representation [50] while representation of 50 to 60 data elements per feature would be a good resolution. For a size range of a bone measuring $\sim 4.5$ to $\sim 21$ mm, a PMCT of 0.3mm resolution would be a theoretical minimum. However, partial volume effects may impede maximally accurate measurements, whereas, counterintuitively, a larger slice thickness of $\sim 3/4$ the object’s size may yield more precise measurement results [51]. While for this study, general forensic PMCT data was evaluated, future PMCT protocols for fetuses and neonates could incorporate specific high resolution reconstructions of relevant body regions, including pars basilaris. For best approximation given the possibilities, this study was performed using thin maximal intensity projection reconstructions upon visualization as explained in Fig. 3 [11]. Overall, its ease of use makes this technique a valuable tool in forensic practice given above mentioned constraints. For easier accessibility of the combined age estimation, we implemented the models in PHP on a freely accessible website^2^.

Future studies may aim to refine the model by expanding its application to different populations, thereby improving its evidence base. Also, correlation studies to more reliably determine bone sizes may become relevant. To better document the method’s technical performance, multi-reader and multi-center studies based on different CT scanners are essential. Additionally, future research could explore the combination of various growth related metrics to identify concurrent or "uncoupled" growth whereas growth and maturation are considered in a differentiated manner [52]. Diagnosis-specific differentiation with regard to fetal development may be a next step in forensic fetal age estimation and diagnosis [53–55].

## Conclusion

In summary, pars basilaris biometry on PMCT was shown to produce age estimates that reliably include true gestational age in a small collective of forensic cases. Integration of ancillary clinical data will further enhance accuracy.

## Keypoints

- This study uses validated statistical methods and a combined method to estimate fetal and infant ages based on the dimensions (width, length) of the pars basilaris of the occipital bone. The underlying model is based on healthy French individuals, the nine Swiss forensic fetuses and newborns that the model was applied to in part had mothers with medical conditions.
- This study combines two separate models from the French study and combines these to a single combined model for practicality.
- Forensic aspects of cases - shrinkage of dry bone in skeletal remains, shrinkage in burnt bodies, unknown maternal conditions - remain a difficulty in accurate age estimation.
- Lack of ancillary information such as maternal health may limit the reliability of a pars basilaris based age estimate.

## Footnotes

1 In [32]: French men: 174,1 cm, French women: 170,1 cm (2001); in [33]: Swiss men 177,6 cm, Swiss women: 164.7 cm (2022).

2 https://www.swisswuff.ch/calculatortest/pb1.php
